# Comparative cost analysis of point-of-care versus laboratory-based testing to initiate and monitor HIV treatment in South Africa

**DOI:** 10.1371/journal.pone.0223669

**Published:** 2019-10-16

**Authors:** Kate Simeon, Monisha Sharma, Jienchi Dorward, Jessica Naidoo, Ntuthu Dlamini, Pravikrishnen Moodley, Natasha Samsunder, Ruanne V. Barnabas, Nigel Garrett, Paul K. Drain

**Affiliations:** 1 Department of Medicine, School of Medicine, University of Washington, Seattle, Washington, United States of America; 2 Department of Global Health, Schools of Public Health and Medicine, University of Washington, Seattle, Washington, United States of America; 3 Centre for the AIDS Programme of Research in South Africa (CAPRISA), University of KwaZulu–Natal, Durban, South Africa; 4 Prince Cyril Zulu Communicable Disease Clinic, Ethekwini Municipality, Durban, South Africa; 5 Department of Virology, National Health Laboratory Service and University of KwaZulu-Natal, Durban, South Africa; 6 Department of Epidemiology, School of Public Health, University of Washington, Seattle, Washington, United States of America; 7 Discipline of Public Health Medicine, School of Nursing and Public Health, University of KwaZulu-Natal, Durban, South Africa; York University, CANADA

## Abstract

**Background:**

The number of people living with HIV (PLHIV) in need of treatment monitoring in low-and-middle-income countries has been rapidly expanding, placing an increasing burden on laboratories. Promising new point-of-care (POC) test have the potential to reduce laboratory workloads, but the implementation cost is uncertain. We sought to estimate the costs of decentralized POC testing compared to centralized laboratory testing for PLHIV initiating treatment in South Africa.

**Methods:**

We conducted a microcosting analyses comparing clinic-based POC testing to centralized laboratory testing for HIV viral load, creatinine, and CD4 count monitoring. We completed time-and-motion studies to assess staff time for sample collection and processing. Instrument costs were estimated assuming five-year lifespans and we applied a 3% annual discount rate. Total costs and cost per patient were estimated over a five-year period: the first year of ART initiation and four years of routine HIV monitoring, following World Health Organization ART monitoring guidelines.

**Results:**

We estimated that per-patient costs of POC HIV viral load, CD4, and creatinine tests were USD $25, $11, and $9, respectively, assuming a clinic volume of 50 patients initiated per month. At centralized laboratories, per-patient costs of POC HIV viral load, CD4, and creatinine tests were USD $26, $6, $3. Total monitoring costs of all testing over a 5-year period was $45 higher for POC testing compared to centralized laboratory testing ($210 vs $166).

**Conclusions:**

POC testing for HIV care and treatment can be feasibly implemented within clinics in South Africa, particularly those with larger patient volumes. POC HIV viral load costs are similar to lab-based testing while CD4 count and creatinine testing are more costly as POC tests. Our cost estimates are useful to policymakers in planning resource allocation and can inform cost-effectiveness analyses of POC testing.

## Introduction

Nearly 37 million people living with HIV (PLHIV) worldwide are either receiving or are eligible to initiate antiretroviral therapy (ART).[1] The World Health Organization (WHO) recommends routine testing of HIV viral load (VL), CD4 count, and creatinine to initiate and monitor patients on ART. In 2015 alone, approximately 10.5 million HIV VL and 20.7 million CD4 count tests were performed in low-and-middle-income countries (LMIC), and annual HIV VL testing is projected to increase to 30 million by 2020.[2] Tests for ART monitoring are generally performed in centralized laboratories, but laboratory capacity is likely inadequate to meet projected future demand,[3–5] in which can result in delays in delivering results and poorer patient health outcomes.[6]

Point-of-care (POC) testing can enable HIV monitoring to be conducted onsite at local clinics, thereby relieving the burden on laboratory facilities, providing rapid delivery of results to patients and providers, and improving linkage to care.[7–9] Further, POC testing can enable expedited detection of ART toxicity and treatment failure resulting in improved treatment outcomes.[10,11] POC testing instruments and differentiated care models are currently being developed and evaluated as promising strategies to improve HIV care.[12–16] However, the cost of implementing POC testing in HIV clinics is uncertain.

South Africa has the largest HIV epidemic in the world, with an estimated 7.9 million PLHIV [17,18]. The large number of HIV cases places a high workload on South African laboratories, which conducted 3.7 million HIV VL tests in 2015 at an estimated cost of $8.7 million.[19] Our objectives in this study were to estimate the total cost of implementing clinic-based POC tests for ART initiation and monitoring in a South African HIV clinic and compare these costs to equivalent tests performed at public centralized laboratories.

## Methods

### Study design

We conducted a detailed microcosting analysis from the clinic perspective to compare the costs of HIV monitoring using decentralized POC tests to the costs of performing equivalent tests at centralized laboratories. Our costing analysis was conducted within the Simplifying HIV TREAtment and Monitoring (STREAM) study, a randomized clinical trial of POC HIV VL monitoring in Durban, South Africa.[20] In STREAM, adult PLHIV who were stable on ART were randomized to receive either 1) POC HIV VL, CD4 count and creatinine monitoring and potential task-shifting to enrolled nurses, or 2) routine laboratory-based monitoring and standard South African HIV care. The STREAM trial was conducted at the CAPRISA eThekwini Research Clinic and the Prince Cyril Zulu Communicable Disease Centre, an urban government clinic providing primary care sexual health, HIV, and TB services. The clinic has both clinical space for patient care where POC creatinine tests are performed by nurses, and a clinic-based POC laboratory where POC CD4 count and HIV VL tests are performed by laboratory technicians.

Ethical approval for this study was granted by the University of Washington Institutional Review Board (STUDY00001466) and the University of KwaZulu-Natal Biomedical Research Ethics Committee (BFC296/16).

### Instrument and materials costs of POC and centralized laboratory testing

We conducted a microcosting analysis to identify materials and instruments used to conduct POC tests and collect samples for both POC and centralized laboratory tests. Materials costs relevant to both POC and laboratory tests included consumables required to collect samples at the clinic and staff salary spent on obtaining blood samples. Blood samples for POC creatinine tests were obtained through capillary finger pricks, while samples for POC CD4 count and HIV VL tests and all centralized laboratory tests were obtained through venous blood draws.

Consumables common to all samples included alcohol swabs, gloves, and adhesive bandages. Samples obtained through blood draws additionally required syringes, needles, and blood tubes. Consumables specific to POC creatinine tests included a lancet and a test strip. Consumables specific to POC HIV VL and CD4 count tests included test cartridges.

### Instrument and maintenance costs of POC tests

This study utilized the following POC testing instruments: Xpert® HIV-1 Viral Load IV (Cepheid, Sunnyvale, USA); Pima^™^ CD4 (Abbott Laboratories, Abbott Park, USA); and StatSensor® Xpress-iTM Creatinine POC (Nova Biomedical, Waltham, USA).

Point-of-care instrument/maintenance costs included: POC test instruments; computers and software; quality control (QC) reagents; refrigerator; and value of laboratory space. We estimated instrument and maintenance costs assuming an instrument lifetime of 5 years, as is common for costing analyses based on WHO guidelines and other published costing literature.[21] Following established WHO costing guidelines which estimate equipment to last an average 5–9 years in low and middle income settings, we assumed POC testing machines have a lifespan of 5 years, which we varied to 10 years in a sensitivity analysis.[22] GeneXpert® instruments are available in a variety of sizes; we examined the costs of a 4-cartridge GeneXpert® instrument, the most common module in use throughout South African laboratories.

### Centralized laboratory costs

Costing data for centralized laboratory tests were obtained from the South African National Health Laboratory Service (NHLS) 2017 price lists. The cost charged to a clinic per test was inclusive of sample transport, laboratory staffing costs, laboratory consumables, instruments, and maintenance. Therefore, for study purposes, centralized laboratory test costs were estimated from the cost charged to clinic per test, plus the cost of professional nurse time and cost of clinic consumables required to collect a sample.

### Time and motion studies

We recorded clinic staff time costs as staff salary per minute multiplied by time spent directly involved with sample collection and/or processing. The following activities were observed:

1. Clinical nursing staff time needed to collect samples (time required to draw blood and complete paperwork).
2. POC test laboratory technician time needed to perform POC HIV VL and CD4 count tests in clinic-based POC test laboratory facility;
3. Clinical nursing staff time needed to perform POC creatinine tests
4. Total time for POC tests to be conducted on their respective instruments, and
5. Time to conduct quality control checks.

We observed 20 patient blood draws to assess time required to obtain blood samples and complete paperwork, and completed 10 individual observations of staff conducting each of the POC creatinine test and the POC HIV VL test (at the time of data collection, POC CD4 count tests were not run at the research facility; however, laboratory technicians reported that the POC HIV CD4 count test required the same hands-on time as the POC VL test, which was directly observed). We computed staff time costs for POC testing by observing the total time needed and removing non-active waiting time during POC test processing as the staff completed routine clinic activities during this time. As staff time required to run tests differed by only a few seconds during the observation period, we averaged observed times to estimate staff time instead of using the range as this would negligibly change the per test cost.

### Analyses

**Fig 1:**
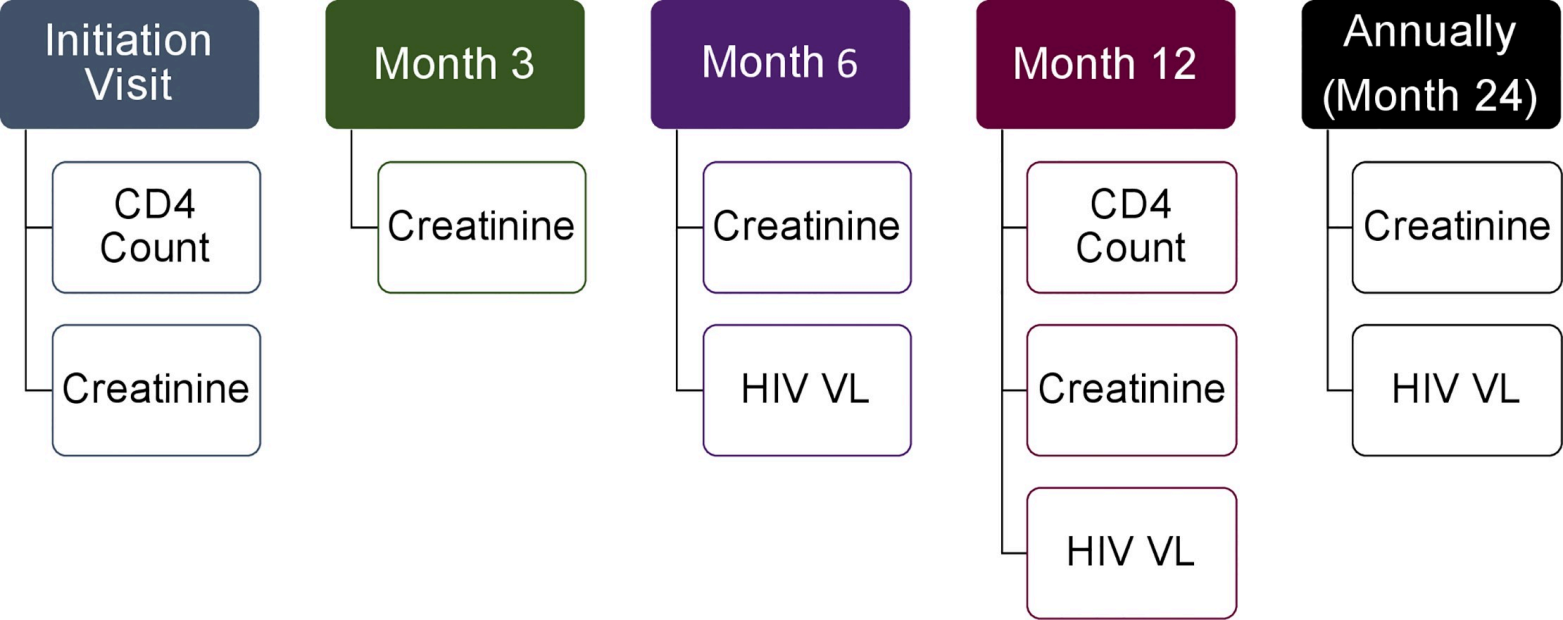
WHO ART monitoring guidelines. Graphical depiction of the various tests required for each visit in the first year of ART initiation as well as each additional monitoring year.

To estimate the total cost of initiating and monitoring a patient on ART for 5 years, we followed the WHO testing and monitoring guidelines to establish the number and types of monitoring tests conducted for one PLHIV over a 5-year period. These guidelines are utilized by the South African Ministry of Health for initiating and monitoring PLHIV on ART (Fig 1):[23]

1. ART Initiation Visit: CD4 Count, Creatinine
2. Month 3: Creatinine
3. Month 6: Creatinine, HIV VL
4. Month 12: CD4 Count, Creatinine, HIV VL
5. Annual Visit After First Year: Creatinine, HIV VL

We assumed the following: the clinic has a stable power supply; POC test material and instrument prices reflect preferential pricing provided by manufacturers for LMICs; patients attended all monitoring visits recommended by WHO guidelines. We did not include utility costs in the POC test costs. Finally, based on consultation with in-country staff, we assumed a typical large South African clinic initiates an average of 50 patients onto treatment per month. However, we varied this assumption in sensitivity analyses. We also conducted a sensitivity analysis excluding creatinine costs since the clinical value of creatinine monitoring is under debate. Further, changes in ART treatment regimens are replacing medications that need creatinine monitoring with new drugs that don’t require routine creatinine monitoring. Finally, we conducted a sensitivity analysis that assumed an instrument lifespan of 10 years.

Staff salary figures and costs of materials and instruments were obtained from published government records, laboratory price lists, clinic invoices, and health economic literature (S1 Table). We calculated the material and staff costs of all POC tests required to initiate and monitor a patient on ART over a 5-year period. POC test instrument costs (instruments, laboratory space value, maintenance costs, and reagent costs) were included by dividing the total 5-year costs by the number of tests performed over the 5-year period. Following established guidelines, capital costs were discounted at 3% annually. Costs were collected in South African Rand and converted to USD using the exchange rate reported at the midpoint of the data collection time period, which took place in July of 2017.[24]

Staff costs including overhead supervision, external quality control checks were not included in our costing estimates. Since the data was collected at a research clinic with a fully functional in-clinic laboratory, it was not possible to estimate the time supervisors spend overseeing tests and how much supervision time would be required in a traditional clinical setting.

## Results

### POC test costs

**Fig 2:**
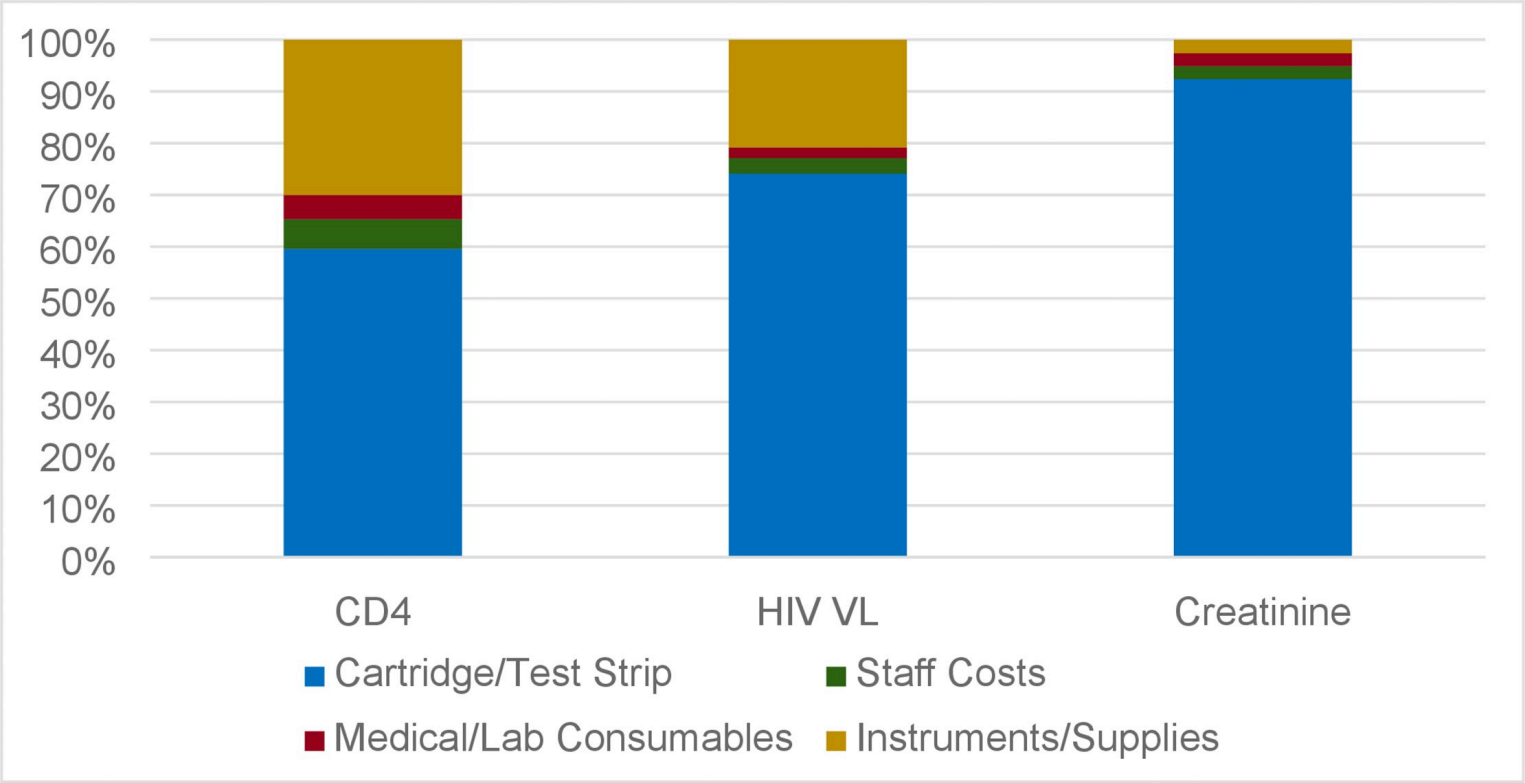
Percentage of total costs per category, assuming clinic load of 50 patients initiated per month. Results: The largest portion of the cost comes from the Cartridge/Test Strip. This category represents 60% of the cost of POC CD4 count, 74% of the costs of POC HIV VL, and 92% of the cost of POC Creatinine.

Individual per-test costs for POC CD4 count, HIV VL, and creatinine tests were estimated to be $11, $25, and $9 respectively, when performed as POC tests at a clinic initiating 50 patients per month for 5 years (Table 1). The largest contributor to the per-test cost was the test cartridge (for POC CD4 count and HIV VL tests) or test strip (for POC creatinine tests), which accounted for 60% of the POC CD4 count test cost, 74% of the POC HIV VL test cost, and 92% of the POC creatinine test cost, assuming a clinic size of 50 patients initiated per month for five years (Fig 2).

**Table 1:** Per-test cost of HIV monitoring tests performed as POC tests and at centralized laboratory facilities in South Africa assuming a patient load of 50 patients initiated each month (2017 US$).

Cost Per Patient	Point of Care		Centralized Laboratory
	5-year POC instrument life	10-year POC instrument life	
**CD4 Count**			
Clinic Medical Consumables	0.41	0.41	0.41
Lab Consumables	0.11	0.11	4.84
Cartridge/Test	6.62	6.62	
Lab Staff Costs	0.20	0.20	
Instrument/Supply Costs*	3.33	1.64	
Clinic Staff Costs	0.44	0,44	0.78
**Total Per Test**	**11.11**	**9.44**	**6.03**
**HIV Viral Load**			
Clinic Medical Consumables	0.41	0.41	0.48
Lab Consumables	0.11	0.11	24.72
Cartridge/Test	18.82	18.82	
Lab Staff Costs	0.33	0.33	
Instrument/Supply Costs*	5.28	1.50	
Clinic Staff Costs	0.44	0.44	0.78
**Total Per Test**	**25.39**	**21.61**	**25.98**
**Creatinine**			
Clinic Medical Consumables	0.22	0.22	0.42
Lab Consumables	0.00	0.00	2.21
Cartridge/Test	8.14	8.14	
Lab Staff Costs	0.00	0.00	
Instrument/Supply Costs*	0.23	0.08	
Clinic Staff Costs	0.22	0.22	0.78
**Total Per Test**	**8.81**	**8.66**	**3.41**

* Assuming a patient volume of 50 pts initiating treatment per month.

Costs related to the value of laboratory space was incorporated equally into total cost of “Instrument/Supply Costs for each type of test”.

The combined cost of instruments, clinic space, and annual maintenance for decentralized POC HIV VL, CD4 count, and creatinine testing over a 5-year period was estimated to be $36,414. After discounting costs pf capital costs, the total cost of increases to $72,084 over a 5-year period. The largest contribution to the cost was the GeneXpert® instrument, at $21,762 (Table 2). Total undiscounted costs per test are reported in S2 Table.

**Table 2:** Costs for establishing and maintaining a clinic-based POC laboratory facility over a 5-year period (2017 US$).

Instruments/Supplies	Initial Fixed Costs	Variable Costs Over 5 Years	Total Cost Per Month
Pima^™^ CD4	7757.65	--	129.29
GeneXpert® Xpert® HIV-1 Viral Load IV	21761.69	--	362.69
StatSensor® Xpress-iTM Creatinine	656.55	--	10.94
Centrifuge	129.04	--	2.15
Refrigerator	132.81	--	2.21
Value of space	2486.11	--	41.44
Pima^™^ CD4 maintenance	--	256.68	5.35
GeneXpert® maintenance	--	2351.28	48.99
Pima^™^ CD4 QC reagents	--	387.64	8.08
StatSensor® Xpress-iTM Creatinine QC reagents	44.31	177.24	3.69
**Total**	32,968.16	3,172.84	602.35
**Discounted Costs**		**35,943.07**	
**Grand Total**		**72,084.07**	

These costs are not dependent on clinic size or number of tests performed.

Discounted costs assume a 5 year life-span for POC machines discounted at 3% annually.

### Sensitivity analyses

As the equipment component of the total test cost per depends on how many tests are conducted, we calculated the cost per patient at varying patient volumes. The cost of initiating and monitoring one PLHIV on ART for a total of 5 years was estimated to be $349 for a clinic initiating 10 patients per month, $211 for a clinic initiating 50 patients per month, and $193 for a clinic initiating 100 patients per month compared to $166 for centralized laboratory testing at any clinic size (Table 3). Patient volumes used in this analysis are limited by the number of tests that can be performed by a single instrument in one day. The GeneXpert® Xpert® IV instrument is the limiting factor as it approaches its maximum capacity at 5 years with 100 patients initiated each month and then followed over this 5 year period (Table 4).

**Table 3:** Sensitivity analysis: Cost to clinic per ART patient initiated and monitored over 5 or 10 years with varying patient volumes (2017 US$).

Point of Care Test					Centralized Laboratory Test
**Number of patients initiated per month**	10	20	50	100	--
**Cost per patient initiated and monitored for a total of 5 years (including HIV VL, CD4 count, and Creatinine)**	349.34	262.71	210.84	193.46	165.83
**Cost per patient initiated and monitored for a total of 10 years (including HIV VL, CD4 count, and Creatinine)**	414.46	367.60	338.86	--	312.78
**Cost per patient initiated and monitored for a total of 5 years (excluding Creatinine)**	281.37	198.73	149.17	132.63	141.96
**Cost per patient initiated and monitored for a total of 10 years (excluding Creatinine)**	307.82	262.24	234.94	--	271.86

Total test cost over 5 years assumes the following tests are conducted for each patient according to WHO guidelines: 2 CD4 count tests, 5 HIV viral load tests, 7 creatinine tests.

Total test cost over 10 years assumes the following tests are conducted for each patient according to WHO guidelines: 2 CD4 count tests, 10 HIV viral load tests, 12 creatinine tests.

**Table 4:** Maximum number of each test performed in a year at various clinic loads.

Number of patients initiated per month		10	20	50	100
**Maximum tests conducted per year over 5 year machine lifetime**	Creatinine	840	1680	4200	8400
	CD4 Count	240	480	1200	2400
	HIV Viral Load	600	1200	3000	6000
**Maximum tests conducted per year over 10 year machine lifetime**	Creatinine	1440	2880	7200	14400
	CD4 Count	240	480	1200	2400
	HIV Viral Load	1200	2400	6000	---

GeneXpert® Xpert® IV has a maximum theoretical capacity of 6000 tests per year at peak efficiency of 24 tests run every day. Five runs of 4 tests can be completed with the 6^th^ run of four started at the end of the day and resulting by the morning. The Pima^™^ CD4 machine also has a maximum theoretical capacity of 6000 tests per year. The StatSensor® Xpress-iTM Creatinine machine has a maximum theoretical capacity of 15000 tests per year.

Assuming creatinine testing is removed from the testing schedule, the cost of initiating and monitoring one PLHIV on ART for 5 years using POC tests is estimated to be $281 for a clinic initiating 10 patients per month, $149 for a clinic initiating 50 patients per month, and $132 for a clinic initiating 100 patients per month (Table 3).

We also conducted a sensitivity analysis assuming a longer instrument lifespan of 10 instead of 5 years. Under this scenario, the cost of initiating and monitoring one PLHIV on ART for a total of 10 years was estimated to be $414 for a clinic initiating 10 patients per month and $339 for a clinic initiating 50 patients per month (Table 3). With a clinic load of 100 patients added per month, the highest number of tests performed in a month exceeds the maximum theoretical capacity of a GeneXpert® Xpert® HIV-1 Viral Load IV and was therefore excluded.

**Fig 3:**
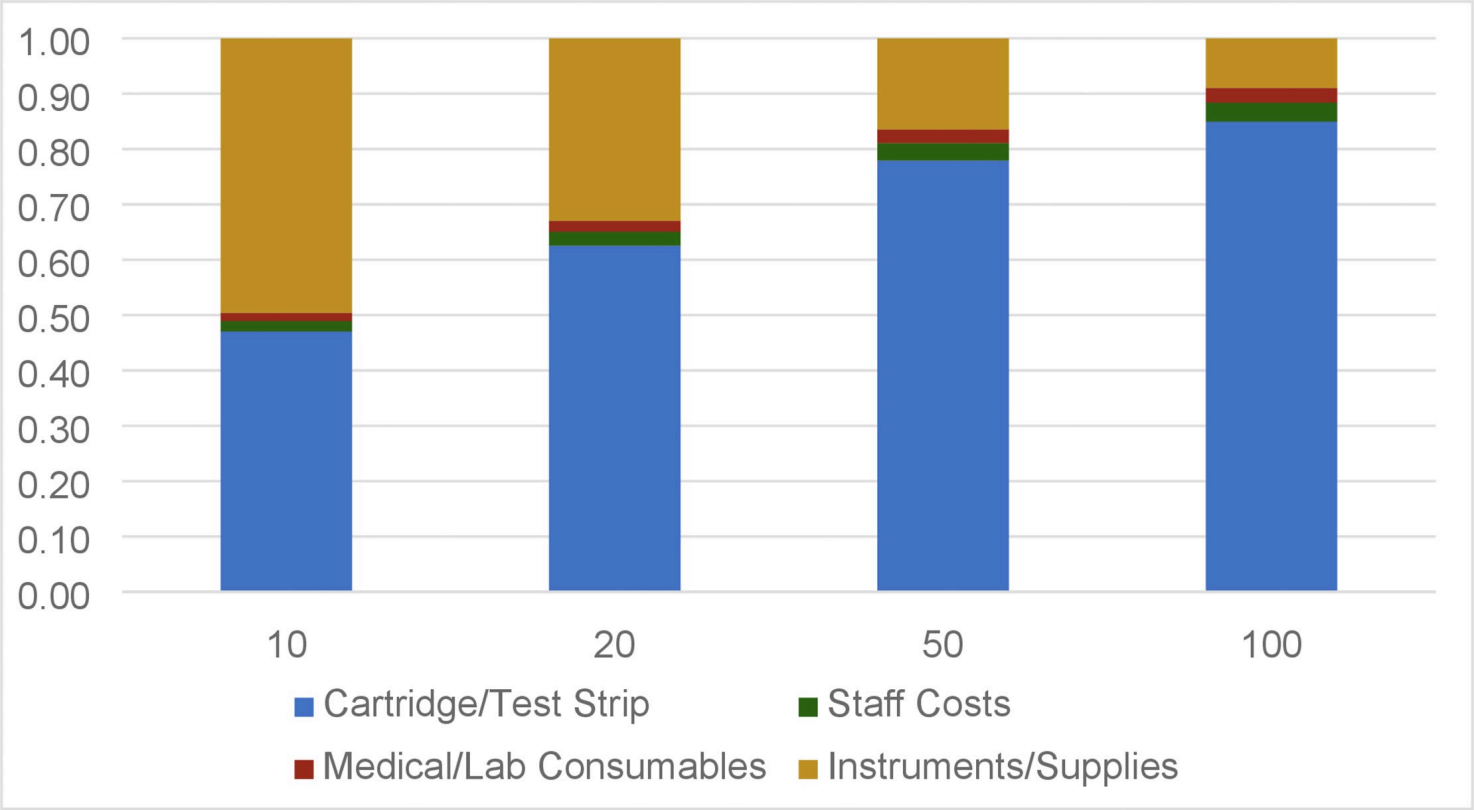
Percent contribution of each cost category to total costs of monitoring over 5 years at various clinic sizes. The largest portion of the total cost at all clinic patient loads is the Cartridge/Test Strip. As patient loads increase and Instrument/Supplies percentage per patient decreases, Cartridge/Test Strip cost becomes a higher percentage of the total cost.

For a 5-year instrument lifespan, instruments costs accounted for 50% of total cost for a clinic initiating 10 patients per month and 16% of total cost for a clinic initiating 50 patients per month (Fig 3).

### Centralized laboratory testing costs

Per-patient costs for CD4 count, HIV VL, and creatinine tests were found to be $6, $26, and $3, respectively, when performed at a centralized laboratory (Table 1). The total cost of initiating and monitoring one PLHIV on ART for 5 years at a centralized laboratory was calculated at $166. The cost of centralized laboratory tests is inclusive of all instrument costs; therefore, we assumed that variations in patient load would not affect the cost charged to clinics by centralized laboratories. If creatinine testing was excluded, the cost of initiating and monitoring one PLHIV on ART for 5 years was estimated to be $142. When assuming a 10-year instrument lifespan, total cost for initiating and monitoring one PLHIV on ART for 10 years at centralized laboratory was calculated at $313 and $272 when excluding creatinine.

## Discussion

In this microcosting study, we estimated the annual cost of implementing POC tests for ART monitoring to be approximately $45 higher per patient compared to using centralized laboratories over a 5-year period. The per-test cost of implementing POC VL testing was approximately the same as centralized laboratories while POC CD4 count and POC creatinine testing $5 and $5 higher per test respectively, compared to laboratory testing. These cost estimates assume a clinic initiates 50 patients per month, which is typical for moderate-size clinics in South Africa. At lower clinic volumes, POC testing became more expensive; for a clinic initiating 10 patients per month, POC testing would be about $183 more expensive per patient over 5 years. These costs include a 3% discount on all instruments and lab space. Discounting costs were not included in centralized laboratory costs as these individual costs were not evaluated in our study. Therefore, it is likely that total equipment costs in this study are a conservative estimate. Overall, we find that POC HIV VL testing can be feasibly implemented into routine care at reasonable costs with minimal processing time for healthcare staff.

Per-patient POC testing costs were highly sensitive to the number of tests performed on each instrument. This is affected by clinic volume and lifespan of instruments. While HIV VL POC costs were comparable to centralized laboratory costs when implemented at moderate to high volume facilities, they were substantially higher than centralized laboratory costs at lower volumes, (e.g. 10 patients initiated on ART per month). Therefore, facility size will likely be an important consideration as countries decide how to scale up POC technology. Costs were also much lower when an instrument lifespan of 10-years and were comparable to centralized laboratory testing.

Creatinine testing was a significant contributor to total POC testing costs, as it is more than twice as expensive to perform as a POC test compared to a centralized laboratory. However, recent evidence suggests that testing creatinine levels in asymptomatic patients on treatment regimens such as tenofovir disoproxil fumarate has limited patient care benefits in low-resource settings.[25] Furthermore, tenofovir alafenamide, which does not require creatinine monitoring, is projected to replace older treatments, reaching 8 million people in LMICs by 2025.[26] Removing creatinine testing from the ART monitoring schedule, or limiting creatinine testing to the first year of ART would decrease POC testing costs to a greater degree than centralized laboratory costs, and could render POC testing costs less expensive in comparison to centralized laboratory testing. Based on previous research, this could be done without risking poor patient outcomes. In our analysis, we found that in the absence of creatinine testing POC monitoring costs were comparable to lab-based monitoring except at very low clinic volumes.

Our cost estimates are similar to those of previous studies.[27,28] Factors that can increase the cost of POC testing include larger amounts of staff time for conducting tests[10] and settings where few tests are conducted such as small clinics.[29] A study conducted in South Africa in 2012[10] estimated the cost of POC CD4 count tests at $19.57 to $23.01, assuming 15 and 5 tests were conducted per day, respectively (prices adjusted to 2017 dollars for comparison to our study). Centralized laboratory-based CD4 count testing was estimated at $7.50 - $8.50 per test, a significantly lower cost. However, this study included the total time for instruments to run a POC test in its cost calculation of total staff time (approximately 20 minutes), in addition to the time required for collecting and processing samples. Staff time contributed $8 to the cost of each test. This method of calculation overestimates the total cost of testing as it is unlikely that nurses would wait for tests to be completed before engaging in other clinical tasks. Because we completed detailed time and motion observations, we attributed only direct hands-on staff time preparing a test sample to total POC testing costs, resulting in more accurate measurements of resource utilization. Another analysis conducted in Zimbabwe, which examined POC testing in infant diagnosis showed that POC testing was cost-effective, provided at least 1 POC test was performed every two days.[29]

An important consideration impacting POC testing costs in South Africa is the potential for using GeneXpert® instruments in clinics to test for TB and sexually transmitted infections, in addition to HIV VL POC testing. Investment in the GeneXpert® instrument would be shared among more patients, improving the potential cost savings of integrating POC testing into clinics and increasing health benefits to patients from TB screening. Since the GeneXpert® instrument is the largest single instrument cost considered in this analysis, this can significantly reduce POC testing cost particularly in clinics with smaller patient volumes which may not be using the instruments at capacity and who would benefit most from increasing the volume of tests performed on instruments.

Additionally, the GeneXpert® instrument is available in varying sizes (sizes include 1 slot, 2 slots, 4 slots, and 16 slots and the Infinity models with 48 and 80 slots); this flexibility can allow clinics to scale the instrument size and corresponding costs to their needs, potentially reducing the cost per POC test. Finally, the costs of instruments for this study were based on prices quoted to a research facility. Manufacturers often offer reduced costs for LMIC and public institutions. For example, a WHO report on the implementation of the GeneXpert® instrument for TB testing estimated the installation costs of the GeneXpert® instrument, combined with annual maintenance costs, to be about $21,700 over 5 years, approximately $2,400 lower than the 5-year costs estimated in this study.[30] Another large component of POC test cost is the test strip or cartridge used in each test, which contributes between 70% and 94% of real total test cost. Large-scale use of POC testing could allow negotiation of a lower cost for test strips and cartridges, decreasing total monitoring costs. Studies show that laboratory supplies and services represent a 6–38% portion of the treatment cost for monitoring ART for PLHIV, highlighting another potential focus for increasing efficiency.[31,32]

There are several strengths and limitations to this study. First, this study does not include the costs of scaling up implementation to a regional or national scale. For example, administrative costs to running POC programs may not contribute significantly to per test costs, they will add significant costs when considering large-scale implementation. Further research will be required to estimate these additional costs. Second, this study was carried out in a single research facility which employed dedicated laboratory and staff to perform POC HIV VL and CD4 count tests, and our results may not be representative of clinics without specialized staff and facilities. In settings where staff have less training, time needed to conduct laboratory testing may be longer, environmental conditions in some clinics may be less ideal for these instruments causing more wear and tear which can shorten their life-span. Finally, we may not have captured all relevant costs: for example, the costs of POC test cartridge disposal (See S3 File: Toxic Material Disposal). A key strength in our analysis was the level of detail included in microcosting and time-and-motion data collection. Additionally, we incorporated time costs for hands-on staff time instead of instrument run time; we assessed a comprehensive POC testing monitoring strategy including all three tests included in the WHO’s HIV-monitoring guidelines, meaning patients would not need to return to clinic for review of any routine laboratory results; and we conducted a range of sensitivity analyses that provide estimates of POC test cost by varying factors including clinic size, changes in instrument and consumable costs, and error rate.

POC testing could provide greatest benefits in rural settings, which also have lower patient volumes, as these locations have decreased access to centralized laboratories and high sample transport costs. Variations in rural clinic conditions, access to utilities, and staff training may also affect factors including the number of tests able to be performed per day, testing error rates, and instrument lifespan. All of these variables affect the volume of tests performed on the instruments and therefore the cost per test at smaller facilities.

In a study conducted in Zambia, researchers examined using POC HIV VL testing in rural settings. They used various methods of service delivery including placing POC testing instruments in clinics, at locations central to multiple rural sites (“POC Hubs”), and a combination of the two. Using these different formats for POC testing delivery, tailored to the needs of the region, they concluded that POC testing was able to reduce the costs of HIV monitoring testing by 6–35%. This was primarily accomplished by reduction in transportation costs and better instrument utilization. [33]

Our study similarly found that POC testing per test costs were highly variable based on number of tests performed on an instrument. Removing inefficiencies and equipment sharing between clinics appears to help decrease costs in smaller, more remote clinics unable to absorb the costs of equipment individually. Finally, while we did not evaluate costs related to increasing distance from centralized laboratories, this will likely be an important cost factor in rural clinics as recent research has shown that transportation costs represent up to 34% of the costs of HIV VL monitoring testing transported to centralized laboratories. [34]

Our study shows that POC technology has the potential to be a financially viable method for clinics with moderate to large patient loads. However, At smaller clinic loads, the upfront costs of instruments and their maintenance makes POC testing more expensive compared to centralized laboratory testing. Further research is needed to evaluate POC testing in non-urban settings, as well as non-research settings. Future microcosting studies of centralized laboratory costs can provide a more detailed understanding of unit cost components compared to our analysis which utilized published costs estimates. In addition, studies are needed to project the cost-effectiveness of POC testing across various clinic volumes. Point-of-care testing offers an opportunity both to improve patient access to testing and monitoring services at reasonable costs.

## Supporting information

S1 File Information about POC instrument costs. (PDF)

S2 File Calculations for staff costs. (PDF)

S3 File Toxic material disposal. (PDF)

S1 Table Assumptions and data sources for centralized laboratory tests and individual cost components of POC tests. An outline of the assumptions and data sources used for each category of costs.
(PDF)

S2 Table Per-test and per-patient cost of HIV monitoring tests performed as POC tests at various clinic loads, not including discounting costs. A summary of per test and per patient costs as described in the manuscript but without discount costs added.
(PDF)
