# Supplementary material for: Comparative cost analysis of point-of-care versus laboratory-based testing to initiate and monitor HIV treatment in South Africa
Source: PLoS One. 2019 Oct 16;14(10):e0223669. doi: 10.1371/journal.pone.0223669 (PMC6795460; doi:10.1371/journal.pone.0223669)
Supplement: S1 File — (PDF) [file pone.0223669.s001.pdf]

S1 Information about POC instrument costs

POC CD4 count instrument/maintenance costs included the Pima™ CD4 instrument, quality control (QC) reagents, refrigerator to house QC reagents, and value of laboratory space. POC HIV VL instrument/maintenance costs included the GeneXpert® HIV-1 viral load instrument (computer, software, and printer included as a package from the manufacturer) and value of laboratory space. POC Creatinine instrument/maintenance costs included the StatSensor® Xpress-i™ Creatinine POC instrument and QC reagents.
