# Supplementary material for: Comparative cost analysis of point-of-care versus laboratory-based testing to initiate and monitor HIV treatment in South Africa
Source: PLoS One. 2019 Oct 16;14(10):e0223669. doi: 10.1371/journal.pone.0223669 (PMC6795460; doi:10.1371/journal.pone.0223669)
Supplement: S2 File — (PDF) [file pone.0223669.s002.pdf]

S2 Calculations for Staff Costs

Yearly salaries used for calculations included $12,243 for lab technicians and $22,647 for professional nurses. To calculate the cost per minute for each profession, each salary was divided by total minutes worked per year. Staff were assumed to work an average of 2016 hours per year. This became $0.10 per minute for lab technicians, and $0.19 per minute for professional nurses.
