# Supplementary material for: Comparative cost analysis of point-of-care versus laboratory-based testing to initiate and monitor HIV treatment in South Africa
Source: PLoS One. 2019 Oct 16;14(10):e0223669. doi: 10.1371/journal.pone.0223669 (PMC6795460; doi:10.1371/journal.pone.0223669)
Supplement: S3 File — (PDF) [file pone.0223669.s003.pdf]

S3 Toxic Material Disposal

The POC HIV VL cartridges contain guanidine thiocyanate: a toxic material used as a lysis reagent that must be disposed of using high-temperature incineration (850 degrees C). This process is expensive and may present extra costs for clinics lacking easy access to these specialized incinerators.[31]
