# Supplementary material for: Comparative cost analysis of point-of-care versus laboratory-based testing to initiate and monitor HIV treatment in South Africa
Source: PLoS One. 2019 Oct 16;14(10):e0223669. doi: 10.1371/journal.pone.0223669 (PMC6795460; doi:10.1371/journal.pone.0223669)
Supplement: S1 Table — An outline of the assumptions and data sources used for each category of costs. (PDF) [file pone.0223669.s004.pdf]

S1 Table 1. Assumptions and data sources for centralized laboratory tests and individual cost components of POC tests

Cost Component	Assumptions	Data Source
**Medical Consumables**	Includes non-reusable items required to collect blood samples from patients. Excludes sharps/waste containers, toner, and paper, as these costs were negligible.	Organization's invoices
**POC Test Supplies**	Includes POC creatinine test strips and POC HIV VL and POC CD4 count test cartridges. Prices include an NGO discount.	Quotes from medical supply companies
**POC Instrument Costs**	Uses prices available to NGOs. Each instrument was assumed to have a 5-year lifetime. Required lab space was estimated, and cost calculated from standard commercial floor space value. Assumes clinic already has stable electricity access and backup.	Quotes from companies, excluding centrifuge and refrigerator: estimated based on quotes from various suppliers
**POC Recurrent Costs**	Includes maintenance and QC materials. Electricity costs and waste per test was assumed to be negligible towards total cost.	Quotes from companies
**Personnel**	Calculated as time spent directly participating in obtaining sample or performing test.	Calculated from government sources, direct observation of activities, and staff interviews
**Centralized Tests**	Based on NHLS quoted prices and assumed to include overhead, transportation costs, staff costs, etc.	NHLS 2017 price list quotes from a local private sector laboratory
