# Supplementary material for: Comparative cost analysis of point-of-care versus laboratory-based testing to initiate and monitor HIV treatment in South Africa
Source: PLoS One. 2019 Oct 16;14(10):e0223669. doi: 10.1371/journal.pone.0223669 (PMC6795460; doi:10.1371/journal.pone.0223669)
Supplement: S2 Table — A summary of per test and per patient costs as described in the manuscript but without discount costs added. (PDF) [file pone.0223669.s005.pdf]

S2 Table 2. Per-test and Per-patient cost of HIV monitoring tests performed as POC tests at various clinic loads, not including discounting costs.

Number of Patients Initiated per Month	10/ Month	20/ Month	50/ Month	100/ Month
**CD4 Count**				
Clinic Medical Consumables	0.41	0.41	0.41	0.41
Lab Consumables	0.11	0.11	0.11	0.11
Cartridge/Test	6.62	6.62	6.62	6.62
Lab Staff Costs	0.20	0.20	0.20	0.20
Instrument/Supply Costs 5	8.55	4.27	1.71	0.85
Instrument/Supply Costs 10	4.33	2.17	0.87	0.43
Clinic Staff Costs	0.44	0.44	0.44	0.44
**Total Per Test for 5 years**	**16.33**	**12.05**	**9.49**	**8.63**
**Total Per test for 10 years**	**12.11**	**9.95**	**8.65**	**8.21**
**Total Over 5 Years**	**32.66**	**24.10**	**18.98**	**17.26**
**Total Over 10 Years**	**24.22**	**19.89**	**17.29**	**16.43**
**HIV Viral Load**				
Clinic Medical Consumables	0.41	0.41	0.41	0.41
Lab Consumables	0.11	0.11	0.11	0.11
Cartridge/Test	18.82	18.82	18.82	18.82
Lab Staff Costs	0.33	0.33	0.33	0.33
Instrument/Supply Costs 5	13.93	6.96	2.79	1.39
Instrument/Supply Costs 10	4.15	2.08	0.83	0.42
Clinic Staff Costs	0.44	0.44	0.44	0.44
**Total Per Test for 5 years**	**34.04**	**27.07**	**22.90**	**21.50**
**Total Per test for 10 years**	**24.26**	**22.19**	**20.94**	**20.53**
**Total Over 5 Years**	**170.19**	**135.37**	**114.48**	**107.51**
**Total Over 10 Years**	**242.65**	**221.87**	**209.41**	**205.25**
**Creatinine**				
Clinic Medical Consumables	0.22	0.22	0.22	0.22
Lab Consumables	0.00	0.00	0.00	0.00
Cartridge/Test	8.14	8.14	8.14	8.14
Lab Staff Costs	0.00	0.00	0.00	0.00
Instrument/Supply Costs 5	0.61	0.31	0.12	0.06
Instrument/Supply Costs 10	0.22	0.11	0.04	0.02
Clinic Staff Costs	0.22	0.22	0.22	0.22
**Total Per Test for 5 years**	**9.19**	**8.89**	**8.70**	**8.64**
**Total Per test for 10 years**	**8.80**	**8.69**	**8.62**	**8.60**
**Total Over 5 Years**	**64.35**	**62.21**	**60.92**	**60.49**

**Total Over 10 Years**	**105.65**	**104.30**	**103.50**	**103.23**
**Grand total over 5 years**	**267.20**	**221.68**	**194.38**	**185.26**
**Grand total over 10 years**	**372.52**	**346.07**	**330.20**	**324.91**
**Grand total no Cr 5 years**	**202.85**	**159.47**	**133.46**	**124.77**
**Grand total no Cr 10 years**	**266.87**	**241.77**	**226.70**	**221.68**
